# miR-151a induces partial EMT by regulating E-cadherin in NSCLC cells

**DOI:** 10.1038/oncsis.2017.66

**Published:** 2017-07-31

**Authors:** I Daugaard, K J Sanders, A Idica, K Vittayarukskul, M Hamdorf, J D Krog, R Chow, D Jury, L L Hansen, H Hager, P Lamy, C L Choi, D Agalliu, D G Zisoulis, I M Pedersen

**Affiliations:** 1 Department of Molecular Biology and Biochemistry, Francisco J. Ayala School of Biological Sciences, University of California, Irvine, CA, USA; 2 Department of Biomedicine, Aarhus University, Aarhus, Denmark; 3 Department of Pathology, Aarhus University Hospital, Aarhus, Denmark; 4 Department of Pathology, Vejle Hospital, Vejle, Denmark; 5 Department of Molecular Medicine, Aarhus University Hospital, Aarhus, Denmark; 6 Departments of Neurology, Pathology and Cell Biology, Pharmacology, Columbia University Medical Center, New York, NY, USA

## Abstract

miR-151a and its host gene, focal adhesion kinase, *FAK*, are located in a region of chromosome 8q that is frequently amplified in solid tumors, including lung cancer. Lung cancer is the leading cause of cancer deaths worldwide and metastasis remains the major challenge in battling lung cancer mortality. Here, we demonstrate that miR-151a is overexpressed in non-small cell lung cancer (NSCLC) patient specimens, as compared to healthy lung. In addition, miR-151a overexpression promotes proliferation, epithelial-to-mesenchymal transition (EMT) and induces tumor cell migration and invasion of NSCLC cells. Blocking miR-151a expression using anti-miR-151a approaches significantly reduced NCSLC cell proliferative and motility potential. Furthermore, we determined that miR-151a significantly regulates E-cadherin expression. Finally, functional rescue experiments determined that overexpression of E-cadherin in miR-151a NSCLC cell lines potently repressed miR-151a-induced partial EMT and cell migration of NSCLC cells. In conclusion, our findings suggest that miR-151a functions as an oncomiR in NSCLC by targeting E-cadherin mRNA and inducing proliferation, migration and partial EMT.

## Introduction

Lung cancer has the highest mortality rate amongst human malignancies and is each year liable for 1.5 million deaths worldwide.^1^ Approximately 85% of all lung cancers are non-small cell lung cancer (NSCLC), which develop as a consequence of both genetic and epigenetic alterations in the epithelial cells of the lung.^2, 3^ The overall 5-year survival rate for lung cancer is 15% and only 5% for patients with distant metastatic lesions.^2, 4, 5^ Epithelial-to-mesenchymal transition (EMT) is recognized as an initiating and essential event in the metastatic cascade in which epithelial cancer cells lose polarity, cell-cell contacts, and acquire mesenchymal features that enhance their migratory and invasive properties.^6, 7, 8, 9^ EMT is a complex process involving a broad spectrum of changes at the molecular level, but downregulation of E-cadherin is considered a hallmark of EMT.^10, 11^

MicroRNA (miRs) are a class of small non-coding RNAs (~22 nt), which regulate gene expression mainly by enhancing messenger RNA degradation (mRNAs).^12^ The majority of all protein-encoding genes are subject to miR regulation and miR dysregulation has been found to be a common feature in human malignancies, including lung cancer.^13, 14, 15, 16, 17, 18, 19, 20, 21, 22, 23^ miR-151a and its host gene, focal adhesion kinase, *FAK*, are located in a region of chromosome 8q that is frequently amplified in solid tumors, including lung cancer.^13, 14, 15, 24^ miR-151a is often expressed with *FAK* and functions synergistically, for example, by promoting metastasis in liver and prostate cancer by inhibiting *RhoGDIA*.^24^ Here we show for the first time that miR-151a is overexpressed in primary NSCLC specimens and induces a mesenchymal-like phenotype, as well as enhances the proliferative, migratory and invasive properties of NSCLC cells, by regulating E-cadherin mRNA and protein expression. Thus, we present a novel mechanism of E-cadherin regulation during tumor cell proliferation, migration and partial EMT in NSCLC.

## Results

### miR-151a is overexpressed in NSCLC

**Figure 1:**
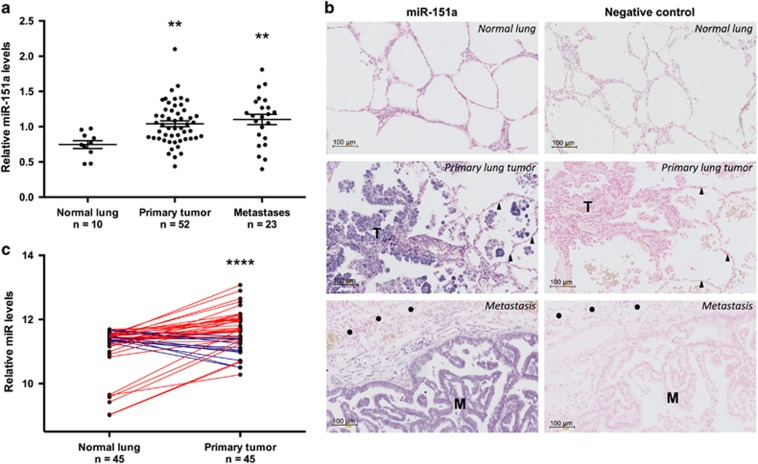
miR-151a expression is induced in NSCLC. (**a**) miR-151a expression levels were characterized by miR-specific RT-qPCR analysis in a NSCLC (LAC) cohort comprising 52 primary LACs and 26 paired distant metastases (22 brain and 4 adrenal gland), as well as 10 tumor-adjacent normal lung samples. Relative expression is shown as a dot plot with mean±s.e.m. in each group indicated. (**b**) *In situ* hybridization using scrambled miR-control and miR-151a probes of normal, primary lung tumor (‘T’) and metastatic (‘M’) tissue. Areas with normal lung and brain cells are indicated with triangles and circles, respectively. High miR expression is shown as blue/purple, low expression is shown as light pink. One representative example shown of 3. (**c**) miR-151a expression in paired tumor and tumor-adjacent normal lung samples from 45 LAC patients. Expression data from all LAC patients with paired specimens available were downloaded from The Cancer Genome Atlas webpage (https://cancergenome.nih.gov/) and log2 transformed. Red or blue lines indicate paired samples with increased or decreased miR-151a expression in the tumor tissue, respectively. **P<0.01, ****P<0.0001; by two-tailed unpaired (**a**) or paired (**c**) Student’s *t*-test.

miR-151a has been shown to function synergistically with its host gene, FAK, which is overexpressed as a result of gene amplification in many types of tumors, including lung cancer, and associated with poor prognosis.^16, 17^ These findings warrant studies of a potential role for miR-151a in NSCLC pathology. We examined miR-151a expression levels in a cohort of 52 patients diagnosed with primary lung adenocarcinoma (LAC), which is the most common subtype of NSCLC.^5, 18, 19^ The cohort comprised 52 primary NSCLCs and 26 matched distant metastases (22 brain and 4 adrenal gland), as well as tumor-adjacent normal lung samples from 10 patients (Supplementary Table S1). We validated that miR-151a is expressed in normal lung, brain and adrenal gland tissue (Supplementary Figure S1) and then performed miR-specific RT-qPCR analysis of all primary NSCLCs, paired distant metastases and tumor-adjacent normal lung samples. miR-151a expression levels were significantly enhanced in primary tumor as compared to normal lung tissue indicating a potential role for miR-151a during NSCLC initiation (Figure 1a, *P*=0.0037). Similarly high expression levels were detected in the paired metastases and the increased expression of miR-151a is thus maintained during the metastatic process. Of note, RT-qPCR analysis was performed on RNA extracted from whole-tissue sections, where the average tumor cell content was 33.2% (range=5–80%, Supplementary Table S1), suggesting that the observed increase in miR-151a expression may be an underestimate. This was validated when we next performed miR-151a *in-situ* hybridization on tissue from paired primary tumor and metastatic sites (brain), as well as tumor-adjacent normal lung, using a scrambled miR control probe as a negative control. Normal lung tissue showed low miR-151a expression levels (Figure 1b). In contrast, miR-151a expression levels were significantly induced in primary tumor, as well as in tissue from the metastatic site (brain) (Figure 1b and Supplementary Figure S2). Finally, we analyzed publicly available microRNA expression datasets through The Cancer Genome Atlas (TGCA) database (https://gdc-portal.nci.nih.gov/). We collected all the hsa-miR-151a expression values from the 45 LAC patients with paired tumor-adjacent normal lung and primary tumor samples available, validating that miR-151a is significantly overexpressed in NSCLC tissue as compared to normal tissue (Figure 1c, *P*<0.0001). These results support the idea that miR-151a functions as a novel oncomiR in NSCLC. We next initiated analysis to mechanistically characterize miR-151a in NSCLC pathogenesis.

### miR-151a enhances NSCLC cell growth

**Figure 2:**
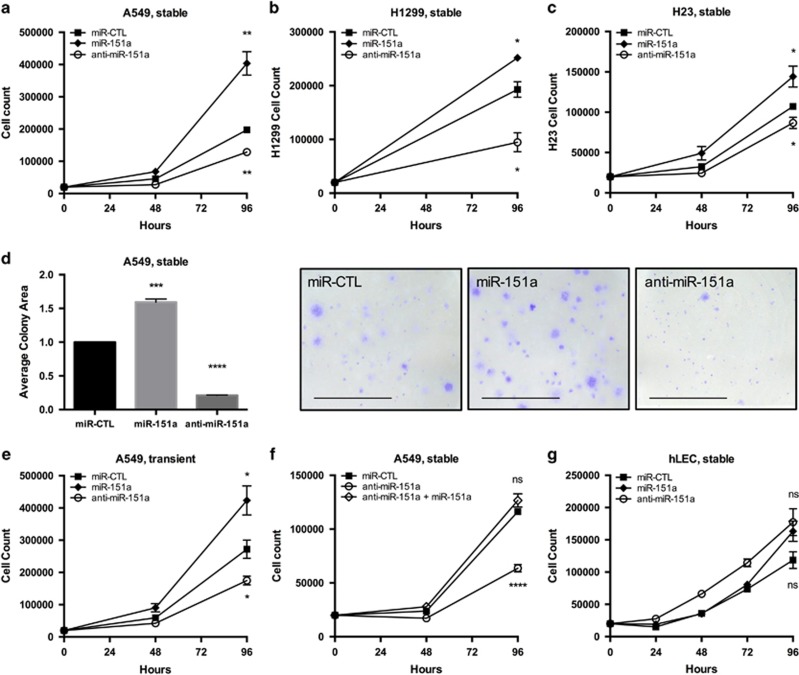
miR-151a enhances NSCLC cell growth. Cell proliferation of miR-modulated (miR-CTL, miR-151a and anti-miR-151a) (**a**) A549 cells, (**b**) H1299 cells and (**c**) H23 cells were analyzed by culturing cells at low density and counted for 4 days. (**d**) Colony Formation Assays of miR-modulated A549 cells. Representative images for each treatment are shown. Scale bar=1000 μm. (*n*=3, independent biological experiments, 3 technical replicates of each). (**e**) Growth analysis counting A549s transfected with miR-CTL, miR-151a or anti-miR-151a oligonucleotides (miR-mimics). (*n*=3 independent cell cultures and experiments, 3 technical replicates for each). (**f**) Growth analysis counting A549s stably expressing miR-CTL, anti-miR-151a or both miR-151a and anti-miR-151a (*n*=3 independent cell cultures and experiments, 3 technical replicates for each). (**g**) Growth analysis counting human lung endothelial cells (hLECs) stably expressing miR-CTL, miR-151a and anti-miR-151a. (*n*=1 biological replicate, 3 technical replicates of each). Throughout figure, all graphs are shown as mean±s.e.m. *P<0.05; **P<0.01; ***P<0.001; ****P<0.0001 by two-tailed Student’s *t*-test.

**Figure 3:**
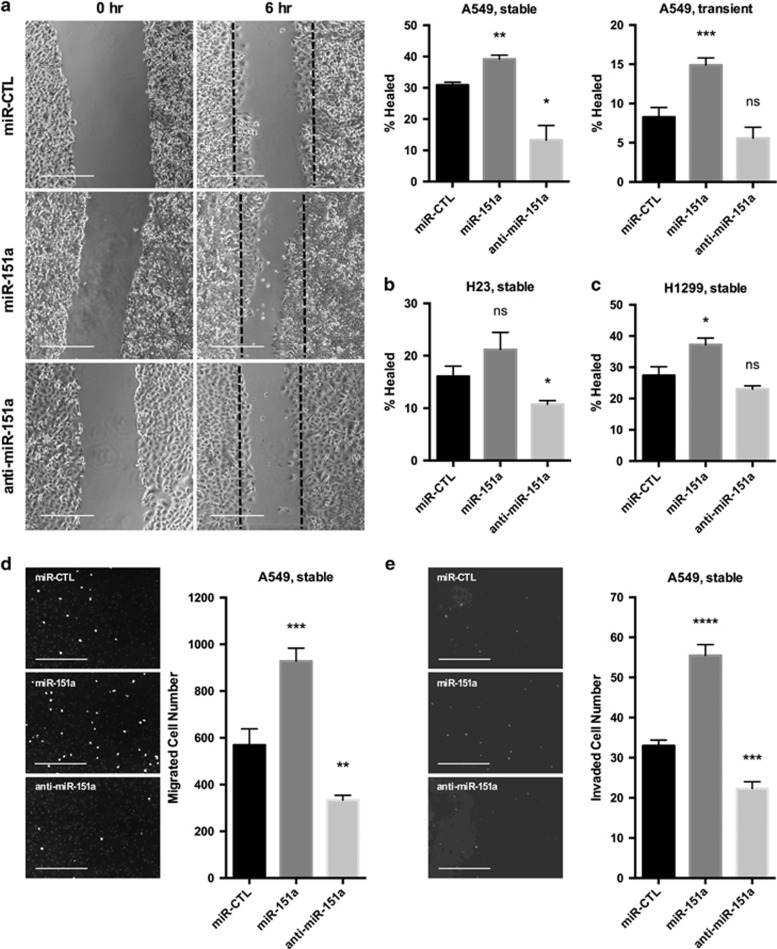
miR-151a enhances NSCLC cell migration and invasion and anti-miR-151a reduces cell motility. (**a**) Stably (Images and left graph) or transiently (right graph) miR-modulated A549 cells (miR-CTL, miR-151a or anti-miR-151a) were pre-treated with mitomycin c for 2 h and then migration was analyzed by scratch assay. Representative images are shown, scale bar=500 μm. (*n*=3 independent cell cultures and experiments). Scratch assays were also performed for stably miR-modulated (**b**) H23 cells and (**c**) H1299 cells. (**d**) Migration of mitomycin c pretreated miR-modulated A549 cells was analyzed by performing transwell migration assays for 6 h, at which point migrated cells were stained with DAPI and counted. Representative images are shown, scale bar=200 μm (*n*=3 independent cell cultures and experiments, 3 technical replicates of each). (**e**) Invasion assay were performed using stable miR-expressing A549s pre-treated with mitomycin c and allowed to migrate for 12 h. Representative images are shown, scale bar=200 μm (*n*=2 independent cell cultures and experiments, 3 technical replicates of each). Throughout figure, all graphs are shown as mean±s.e.m. *P<0.05; **P<0.01; ***P<0.001; ****P<0.0001 by two-tailed Student’s *t*-test.

To investigate if enhanced expression of miR-151a promotes NSCLC cell tumor cell growth and motility, we generated miR-modulated NSCLC cell lines using A549 cells (miR-151a over-expression, anti-miR-151a cell lines in which endogenously expressed miR-151a is neutralized, and control miR cell lines). miR modulated NSCLC cell lines were verified for miR-151a expression levels and effect on a published miR-151a target, RhoGDIA,^24^ (Supplementary Figure S3). miR-151a was determined to be 2–4 fold increased in A549 cells over-expressing miR-151a (miR-151a A549 cells), and 30-50% reduced in anti-miR-151a A549 cells, relative to miR controls. First, we determined that miR-151a overexpression significantly enhanced, whereas anti-miR-151a significantly reduced A549 cell growth, as compared to control A549 cells (Figure 2a, *P*=0.005, *P*=0.0045 and Supplementary Figure S4A). We verified that this was not an A549 cell line specific phenomena, as miR-151a also significantly enhanced H23 and H1299 tumor cell proliferation, and anti-miR-151a significantly decreased tumor cell proliferation, relative to miR control H23 and H1299 cells (Figures 2b and c). These results suggest that NSCLC cells with different genetic aberrations (A549: *p14ARF* and *KRAS* mutations), (H23: *KRAS* and *PTEN* mutations) and (H1299: *NRAS* mutations and *p53-null*) are sensitive to changes in miR-151a expression levels. Next we performed colony-formation assays and obtained comparable results for miR modulated A549 cells (Figure 2d, *P*=0.0003 and *P*<0.0001). In addition, A549 cells were transiently transfected with miR mimic oligonucleotides (miR-151a, anti-miR-151a or miR control mimics). These experiments showed a similar effect on tumor cells growth (Figure 2e, *P*=0.0472 and *P*=0.0366, and Supplementary Figure S4B), demonstrating that the observed effect of stably induced miRs, are not likely to be an artifact of lentiviral genomic integration. Furthermore, we generated an anti-miR-151a/miR-151a double expressing A549 cell line. Adding miR-151a back into anti-miR-151a A549 cells rescued the anti-growth effect, indicating that overexpressed miR-151a is indeed required for anti-miR-151a reduced A549 tumor cell growth (Figure 3e, anti-miR-151a: *P*<0.0001 and Supplementary Figure S4C). Finally, miR-modulated primary human lung endothelial cells, (hLECs) were analyzed, suggesting that the proliferative advantage of miR-151a, and that anti-miR-151a’s cytostatic effects are selective to NSCLCs, relative to the analyzed hLECs (Figure 3g and Supplementary Figure S4D).

### miR-151a increases NSCLC cell motility

Successful tumor cells acquire enhanced migratory and invasive properties. We next tested if miR-151a plays a role in NSCLC cell motility. miR modulated (miR-151a, anti-miR-151a and control miR) cell lines were pre-treated with mitomycin c, a cell cycle inhibitor (Supplementary Figure S5A), allowing us to study effects on migration, separate from proliferation. First the classical wound healing assays was performed. miR-151a A549 cells were capable of healing the induced wound at a significantly increased rate, as opposed to anti-miR-151a A549 cells, which showed significantly reduced wound healing, relative to control miR A549 cells (Figure 3a, **P=0.0059, *P=0.0196 (transduced cells). Similar results were obtained using transfection of miR mimics (Figure 3a, miR-151a: *P*=0.0005 and Supplementary Figure S5B). We tested and verified that miR-151a regulation (miR-151a versus anti-miR-151a) substantially increased versus reduced wound healing, relative to miR control in both H23 cells and H1299 cells (Figures 3b and c, H23: *P=0.0304, H1299: *P=0.0499 and Supplementary Figures S5Cx and D). In addition, we performed transwell migration assays and *in vitro* invasion assays, to further evaluate miR-151a’s regulatory role in NSCLC cell motility. As expected miR-151a A549 cells migrated through the transwells at a significantly increased rate, and anti-miR-151a A549 cells migrated less efficiently, as compared to control miR A549 cells (Figure 3e, ***P=0.001 and **P=0.0053 and Figure 3f, miR-151a: ****P<0.0001, anti-miR-151a: ***P<0.0007). In summary, these results support the idea that miR-151a significantly enhances the potential for NSCLC tumor cell migration and invasion and that neutralization of miR-151a results in a substantial reduction in NSCLC cell motility.

### miR-151a induces a mesenchymal-like morphology of NSCLCs

**Figure 4:**
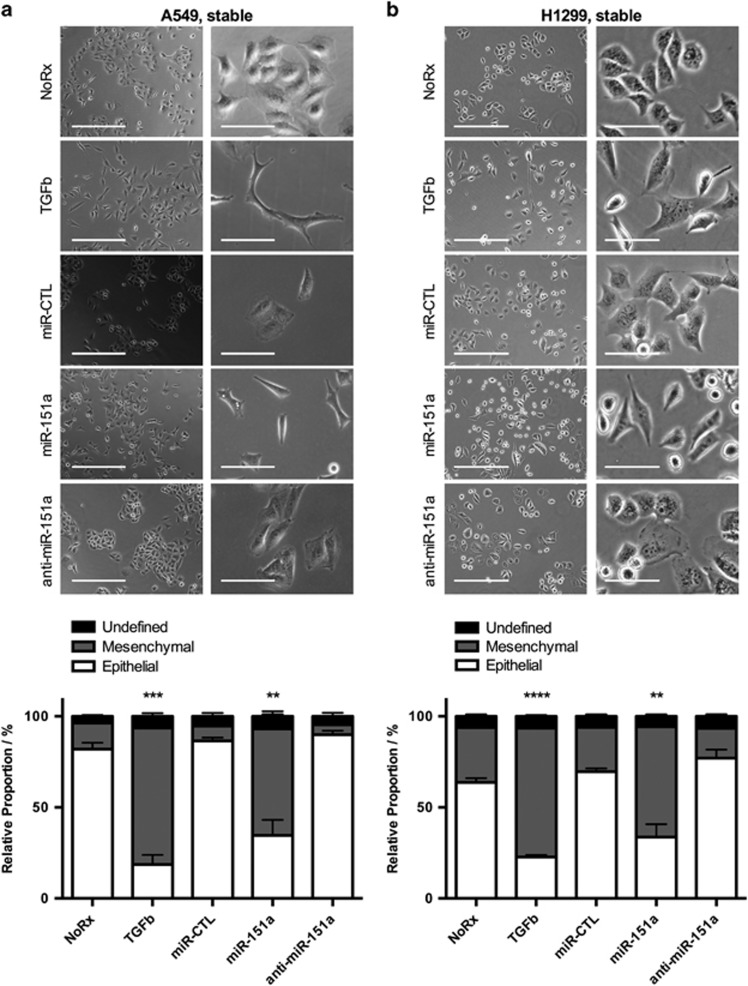
miR-151a induces a mesenchymal-like phenotype and anti-miR-151a enhances an epithelial cell-like phenotype of NSCLC cells. Images (top panels) of (**a**) A549 cells and (**b**) H1299 cells untreated, treated with TGF-β, or stably transduced with miR-CTL, miR-151a or anti-miR-151a. Column 1: Bright field (Scale bar=400 μm), Column 2: Bright field (scale bar=100 μm). The relative proportion of mesenchymal, epithelial, or undefined cells were quantified and shown as stacked bar percentage plots (lower panels). The relative proportion of mesenchymal cells was compared between treatments using two-tailed Student’s *t*-test. One representative image shown of three independent cell cultures and experiments. Throughout figure, all graphs are shown as mean±s.e.m. **P<0.01; ***P<0.001; ****P<0.0001 by two-tailed Student’s *t*-test.

Based on an observed change in tumor cell morphology we next initiated quantitative analysis comparing the morphology of untreated, miR modulated and NSCLC cells stimulated with TGF-β, which is a strong inducer of EMT.^7^ We determined that miR-151a is an inducer of a mesenchymal-like morphology of A549 cells (spindle-formed, far separated cells), relative to untreated and miR control A549 cells, though not to the extent of TGF-β (Figure 4a, miR-151a: mesenchymal=77.7%, epithelial=17.7%, undefined=4.6% and Supplementary Figure S2). Anti-miR-151a cells showed an epithelial-like cell phenotype (cobblestone shaped, cluster formation), similar to that of parental A549 cells (Figure 4a, anti-miR-151a: mesenchymal=6.4%, epithelial=91.6%, undefined=2.0% and Supplementary Figures S6 and S7). Similar results were obtained using miR modulated H1299 cells. Interestingly, in addition to the effect of miR-151a, anti-miR-151a consistently showed an effect on H1299 cells by decreasing the percentage of mesenchymal-like cells, relative to miR control H1299 cells (Figure 4b). These results suggest that miR-151a overexpression induces a conversion of the characteristic epithelial cell phenotype of NSCLC cells (A549 and H1299 cells) to a partial mesenchymal-like phenotype, and suggests that anti-miR-151a can reduce these cellular traits characteristic of tumor cell progression in NSCLC cells (H1299 cells).

### miR-151a reduces E-cadherin in NSCLC cells

**Figure 5:**
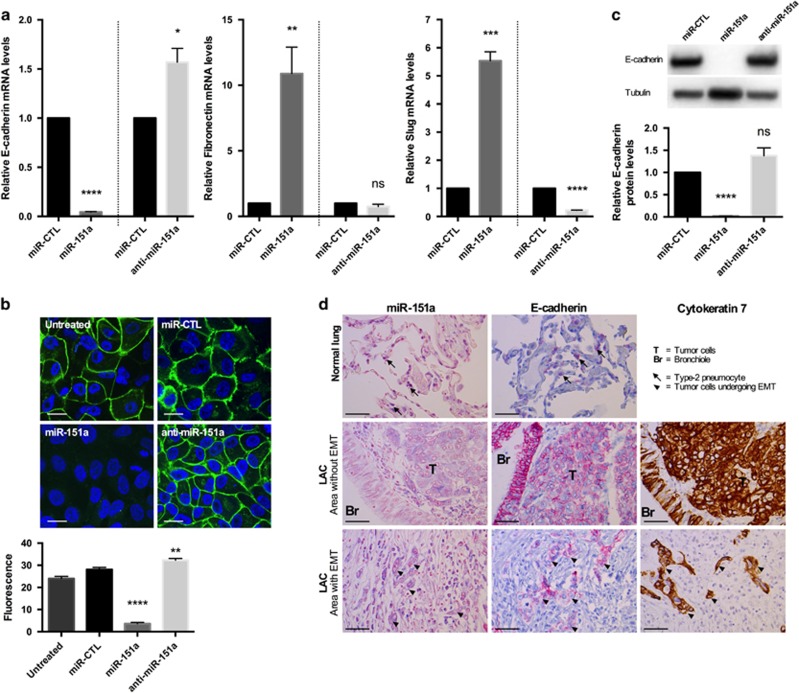
miR-151a reduces E-cadherin expression in NSCLC cells. (**a**) The relative expression levels of E-cadherin (left), fibronectin (middle) and Slug (right) were determined in stably miR-transduced A549 cells by RT-qPCR. (*n*=3 independent cell cultures and experiments, 3 technical replicates of each). (**b**) Representative confocal images of stably transduced A549s immunofluorescently stained with E-cadherin. Scale bar=10 μm. Relative fluorescence levels are quantified of the shown biological replicate (*n*=3 independent cell cultures and experiments, 12 technical replicates of each). (**c**) Immunoblot analysis of E-cadherin and tubulin protein levels in A549s transduced with miR constructs. Relative levels are quantified. One representative example of three is shown. (**d**) The expression levels of miR-151a was analyzed by *in situ* hybridization and compared with the expression of E-cadherin determined by immunohistochemical staining in three normal lung samples and 3 primary LACs (miR-151a expression: high=purple, low=light pink, E-cadherin expression: high=pink, low=blue). Cytokeratin 7 was included to identify cells of epithelial origin, for example, adenocarcinoma cells. Scale bar=50 μm. Throughout figure, all graphs are shown as mean±s.e.m. *P<0.05; **P<0.01; ***P<0.001; ****P<0.0001 by two-tailed Student’s *t*-test. Uncropped version of blot is shown in Supplementary Figure S8.

Our finding that miR-151a induces a mesenchymal-like morphological phenotype and enhances NSCLC cell migratory potential (Figures 3a and 4), suggests that miR-151a induces partial EMT of lung cancer cells. Following up on these findings we next performed RT-qPCR analysis of miR-modulated A549 cells and found that miR-151a overexpression resulted in significantly induced expression levels of Fibronectin and Slug mRNA (Figure 5a, *P*=0.008 (fibronectin) and *P*=0.0001 (Slug)), and anti-miR-151a resulted in reduced expression levels of Fibronectin and Slug mRNA, relative to controls (Figure 5a, *P*<0.0001 (Slug)). It has previously been determined that Slug represents one of the major master regulators of EMT in lung cancer.^20^ In addition, miR-151a significantly reduced the expression levels of E-cadherin and anti-miR-151a enhanced E-cadherin mRNA expression levels, relative to controls (Figure 5a, *P*<0.0001). Expression levels of Snail, Twist and ZEB1 mRNA were not significantly increased (data not shown). Loss of E-cadherin is considered to be a fundamental event in EMT and many transcription factors are known to repress E-cadherin during EMT, including Slug.^6, 7, 8, 9^ We next wished to determine whether miR-151a reduces E-cadherin protein expression levels. Confocal analysis of E-cadherin expression of miR modulated A549 cells showed that overexpression of miR-151a significantly reduced the expression levels of E-cadherin, and anti-miR-151a enhanced E-cadherin protein expression, as compared to control cells (Figure 5b, *P*<0.0001 and *P*=0.0026). Western blot analysis also confirmed a significant effect of miR-151a on E-cadherin protein levels and a similar trend was seen for anti-miR-151a (Figure 5c, miR-151a: *P*<0.0001, anti-miR-151a: *P*=0.1017). Next, we wished to study E-cadherin expression in NSCLC patient specimens. First we determined E-cadherin expression levels in the 52 primary NSCLC samples by RT-qPCR using RNA extracted from whole tissue sections, and compared the levels to those determined for miR-151a in each sample (Figure 1a). No inverse correlation between miR-151a and E-cadherin expression was detected (Supplementary Figure S9A), possibly because of the low and varying percentages of tumor tissue in the samples (mean=33.2%, range=5–80%, Supplementary Table S1). Next we performed immunohistochemical analysis of E-cadherin expression in normal lung and primary NSCLC tumor (Figure 5d). We were surprised to find that both miR-151a and E-cadherin showed strong positive staining in NSCLC (Area without EMT), as compared to normal lung (Figure 5d). When comparing E-cadherin and miR-151a expression between areas within the same slide where EMT was absent versus had occurred, we were able to identify areas which showed the expected inverse correlated between miR-151a and E-Cadherin (high versus low). However, the variation in both miR-151a and E-cadherin staining intensity across and between slides rendered direct comparison inappropriate. We also evaluated the expression of E-cadherin and miR-151a in the NSCLC area without EMT (Figure 5d), and interestingly we observed that miR-151a expression was higher in the tumor tissue (‘T’) as compared to the epithelial cells in the adjacent bronchiole (‘Br’), which represents normal lung tissue. The inverse relationship was seen for E-cadherin, which was expressed at higher levels in the bronchiole (‘Br’) as compared to the tumor cells (‘T’). This is an important observation for two reasons, (1) the bronchiole and tumor tissue were immediately adjacent to each other and the differences in miR-151a and E-cadherin expression between the bronchiole and tumor tissue was therefore not a result of variation in staining intensity across the slide and (2) both epithelial cells from the bronchiole as well as the NSCLCs are believed to derive from the same stem cells within the lung epithelial cell tissue.^21^ However, further analysis is needed to demonstrate whether miR-151a is regulating E-cadherin expression *in vivo*.

### miR-151a interacts with the coding sequence of E-cadherin mRNA

**Figure 6:**
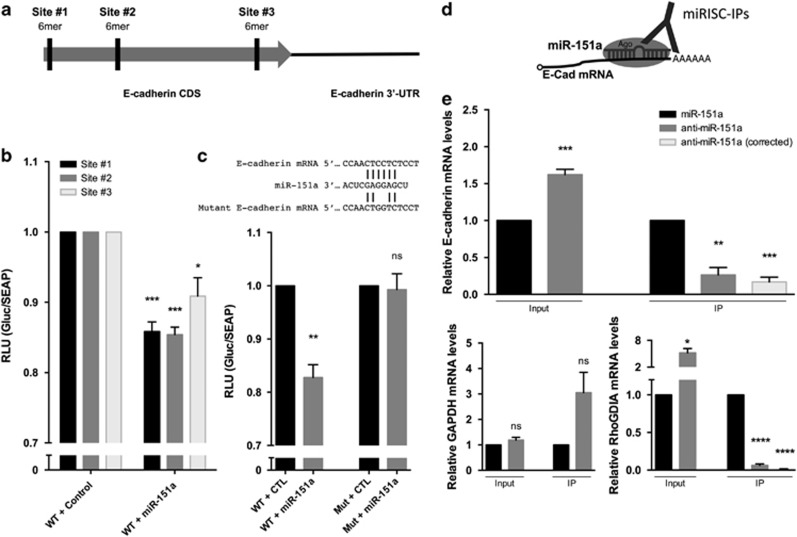
miR-151a interacts with E-cadherin mRNA. (**a**) Schematic representation of three potential 6-mer miR-151a-binding sites in the coding DNA sequence (CDS) of E-cadherin mRNA. (**b**) Relative luciferase levels in A549 cells transfected with constructs expressing the wild type (WT) binding sequences at site #1, #2 or #3, along with miR-CTL or miR-151a mimics, were determined 48 hr after transfection. (*n*=3 independent cell cultures and experiments, 3 technical replicates of each). (**c**) Schematic of miR-151a binding to WT or mutant E-cadherin mRNA. Relative luciferase levels of A549 cells transfected with constructs expressing WT or mutated (site #2) binding sequences and miR-CTL or miR-151a mimics were determined 48 hr after transfection. (*n*=3 independent cell cultures and experiments, 3 technical replicates of each). (**d**) Schematic of Argonaute immunopurification strategy for miR-RNA complexes (Ago-RIP). (**e**) A549 cells stably expressing either miR-151a or anti-miR-151a were generated. Relative levels of E-cadherin, RhoGDIA and GAPDH mRNA in input and Ago-RIP (IP) fractions were determined. ‘Corrected’ indicates mRNA levels in IP fractions when normalized to the mRNA level in corresponding input fractions. (*n*=3 independent cell cultures and experiments, 3 technical replicates of each). Throughout figure, all graphs are shown as mean±s.e.m. *P<0.05; **P<0.01; ***P<0.001; ****P<0.0001 by two-tailed Student’s *t*-test.

We next wished to determine if E-cadherin mRNA is a direct target of miR-151a. When performing bioinformatics analyses of potential miR-151a binding sites in E-cadherin mRNA, we identified the same identical (6-mer) seed match at three different locations in the coding sequence (CDS) of E-cadherin mRNA, named site #1, site #2 and site #3 (Figure 6a). In order to determine whether the predicted 6-mer seed sequence, was required for miR-151a binding we performed a series of E-cadherin luciferase reporter assays, in which fragments of the E-cadherin CDS including either site #1, site #2 or site #3 were cloned into a luciferase reporter construct. HeLa cells were transfected with one of the three E-cadherin CDS-luciferase-encoding plasmids in the presence of either mature miR-151a or miR control mimics. HeLa cells transfected with miR-151a and encoding binding site #1, #2 and #3 all showed modest but significantly reduced luciferase activity (Figures 6a and b, #1 *P*=0.0005, #2 *P*=0.0002, #3 *P*=0.0255). Next, mutations were introduced into the 6-mer seed site of E-cadherin CDS (site #2) to determine if this specific nucleotide sequence is required for the interaction with miR-151a (Figure 6c). Luciferase activity was again significantly lower in HeLa cells transfected with miR-151a mimic and WT E-cadherin, relative to miR control mimic suggesting that miR-151a can bind to the WT E-cadherin mRNA sequence and prevent the translation of luciferase (Figure 6c, *P*=0.0021). In contrast, HeLa cells transfected with miR-151a mimic and the mutant 6-mer E-cadherin CDS site, exhibited de-repressed luciferase activity to the same levels as the WT E-cadherin and miR-control cells; consistent with the idea that miR-151a no longer binds and represses reporter gene expression (Figure 6c).

Next we performed Argonaute (Ago)-miR immunopurification analysis. In brief, Ago complexes containing miRs and target mRNAs were isolated by immunopurification and assessed for relative complex occupancy by the E-cadherin mRNA by qRT-PCR to determine if miR-151a directly targets E-cadherin mRNA in A549 cells (Figure 6d), as previously described.^22^ The relative level of E-cadherin mRNA was significantly lower in cells stably overexpressing miR-151a when compared to those expressing anti-miR-151a constructs, as expected (Figure 6e, *P*=0.0010). Despite the increased levels of E-cadherin mRNA (because of lower miR-151a expression levels), which may underestimate the scale of the effect, the relative fraction of Ago-bound E-cadherin mRNA significantly increased when miR-151a was overexpressed (Figure 6e, IP, *P*=0.0018). When correcting for the lower expression level of E-cadherin mRNA, the increase in miR-151a bound E-cadherin mRNA was even more significant (Figure 6e, IP, *P*=0.0002). In contrast, miR-151a did not repress GAPDH mRNA expression levels or immunepurify GAPDH mRNA, as expected (Figure 6f). As a positive control we immune purified a different miR-151a target mRNA, RhoGDIA.^24^ As expected miR-151a significantly reduced the expression levels of RhoGDIA mRNA, and miR-151a immunepurified significantly more RhoGDIA mRNA as compared to anti-miR-151a A549 samples (Figure 6g Input, *P*=0.0116, IP, *P*<0.0001). These data combined, support the idea that miR-151a represses E-cadherin expression via a direct interaction with the target site on the E-cadherin CDS mRNA. However, further characterization demonstrating whether the proposed sites are functional in the context of the full length coding region, is warranted.

Importantly genomic alignment analysis of human, gorilla, giant panda, leopard, mouse and cat sequences showed that the miR-151a-binding sites in E-cadherin coding region sequence are 100% conserved for site#3 between all 6 species, and that binding site#1 and site#2 are 100% conserved between human and mouse versus human and Gorilla, and partly conserved (one–three mismatches) in the remaining alignment analysis (Supplementary Figure S9B).

### E-cadherin is a functional target important for miR-151a-induced partial EMT

**Figure 7:**
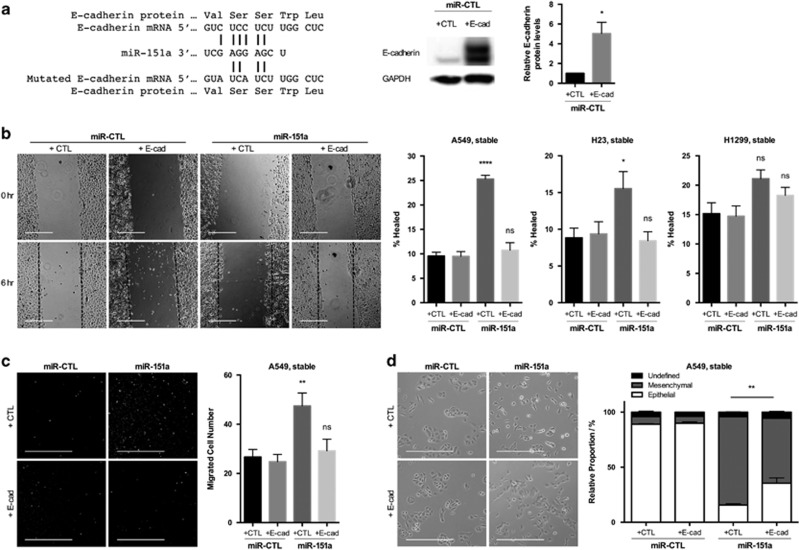
E-Cadherin is a functional target of miR-151a-induced partial EMT. (**a**) Schematic representation showing miR-151a binding to WT or mutant (silent mutant) E-cadherin mRNA (miR-151a resistant E-cadherin, left panel). Immunoblot analysis of E-cadherin and GAPDH protein levels in A549 cells transduced with miR-151a resistant E-cadherin or control constructs (middle panel). Relative levels are quantified (right panel). (**b**) Migration of mitomycin c pre-treated A549 cells (images and left graph), H23 cells (middle graph) and H1299 cells (right graph) transduced with miR-CTL or miR-151a and miR-151a resistant E-cadherin or control constructs were assessed by wound healing assay at time 0 and 6 h. Representative images (left panels), scale bar=500 μm. (*n*=3 independent cell cultures and experiments, 3 technical replicates of each). (**c**) Transwell migration assay using A549s transduced with miR-CTL or miR-151a and miR-151a resistant E-cadherin or control constructs. Cells were pre-treated with mitomycin c, plated at the top of a transwell and allowed to migrate for 6 h. Representative images are shown (left panels, scale bar=400 μm. (*n*=3 independent cell cultures and experiments, 3 technical replicates of each). (**d**) Images of A549 cells transduced with miR-CTL or miR-151a and miR-151a resistant E-cadherin or control constructs. Representative images are shown (left panel), scale bar=400 μm. The relative proportion of mesenchymal, epithelial, or undefined cells were quantified and shown as a stacked bar percentage plot (right panel). The relative proportion of mesenchymal cells was compared between treatments using two-tailed Student’s *t*-test. (*n*=3 independent cell cultures and experiments, three technical replicates of each). Throughout figure, all graphs are shown as mean±s.e.m. *P<0.05; **P<0.01; ***P<0.001; ****P<0.0001 by two-tailed Student’s *t*-test.

As miRs are known to target multiple, even hundreds of mRNAs, we wished to evaluate the significance of E-cadherin as a direct functional mediator of the miR-151a-induced proliferation and partial EMT, including migration. We generated an E-cadherin over-expressing plasmid in which the miR-151a binding site was mutated by creating silent mutations in the 6-mer-binding site (called miR-151a resistant E-cadherin) (Figure 7a, left panel). We verified that E-cadherin was induced in E-Cadherin induced A549 cells relative to A549 control cells (Figure A, middle and right panel). First, we determined that ectopic E-cadherin expression did not specifically rescue miR-151a-induced proliferation (data not shown). Next, we examined the effect of miR-151a resistant E-cadherin, on NSCLC cell migration by performing scratch assay rescue experiments. Ectopic E-cadherin expression reduced miR-151a-induced migration, as determined by wound healing assays of A549, H23 and H1299 miR-modulated cells (Figure 7b, A549: **** *P*<0.0001, H23: * *P*=0.0237). In addition, E-cadherin over-expression in miR-151a A549 cells reduced A549 cell migration as determined by transwell migration assays (Figure 7c, *P*=0.0014). Finally, we analyzed the effect on NSCLC cell morphology and determined that introduction of miR-151a resistant E-cadherin into miR-151a over-expressing cells, partially rescued the effect of miR-151a, as the transition into a mesenchymal-like phenotype was greatly reduced (Figure 7d, ** *P*=0.0094). In summary, we propose a model for oncomiR-151a-induced partial EMT in NSCLC cells, in which miR-151a directly targets the CDS of E-cadherin and represses the expression of E-cadherin protein, resulting in a significant growth advantage and the induction of a mesenchymal-like transition including enhanced motility.

## Discussion

In the present study we demonstrate for the first time that miR-151a functions as an oncomiR in NSCLC. We find that miR-151a is significantly increased in NSCLC tumor specimens as compared to normal lung tissue and that ectopic miR-151a expression significantly enhances NSCLC cell proliferation, but does not significantly affect normal lung endothelial cell growth. In addition, overexpression of miR-151a induces a partial EMT phenotype of NSCLCs cells as determined by a change in cell morphology and significantly enhanced cell motility and invasive properties. The functional characteristics of miR-151a NSCLC cells, correlates with a significant decrease of E-cadherin expression and increases of Fibronectin and Slug expression levels, which are considered fundamental events in EMT. Direct interaction analyses by luciferase reporter assays and miR-Ago immunopurification methods support the idea that miR-151a regulates E-cadherin expression and that miR-151a directly binds to seed sequence matches in the coding region sequence. Importantly, genomic alignment analysis demonstrated sequence conservation of the miR-151a binding sites in the E-cadherin coding region sequence (in particular of site#3) between species.

However, an alternative explanation for the effect of miR-151a on E-cadherin is that the remarkable upregulation of Slug by miR-151a leads to the repression of E-Cadherin transcription.

At this point the mechanism by which overexpression of miR-151a results in significant induction of Slug (and fibronectin) mRNA expression levels is unknown. We are in the process of investigating possible different scenarios, namely whether miR-151a targets a negative regulator of Slug, resulting in the increase in Slug expression. An alternative explanation could be aligned with a previously reported finding by Onder *et al.*,^23^ demonstrating that E-cadherin loss can results in the induction of multiple transcription factors including Twist and Zeb-1 in breast cancer cells (Onder *et al.*^23^). However, the argument that the upregulation of Slug is a consequence, rather than a cause, of E-Cadherin repression is weakened by the fact that neither Zeb1 nor Twist were induced in the NSCLC cell lines studied. As such, the upregulation of Slug merits further investigation. Additional work is also required to address the question as to how miR-151a is regulated and induced. Pilot experiments suggest that TGF-beta does not induce miR-151a expression levels in NSCLC cells.

Furthermore, induced miR-151a resistant E-cadherin overexpression in miR-151a NSCLC cells abrogated the induced partial EMT transition and migration advantage of miR-151a NSCLC cells, indicating that E-cadherin is an important functional target of miR-151a-induced migration. Additional work is needed to determine which miR-151a targets are responsible for the observed NSCLC cell growth advantage. The finding that miR-151a overexpression also significantly induces the expression levels of fibronectin and Slug in A549 cells, suggests that miR-151a may be involved in the regulation of a complex network of proteins involved in EMT. The importance of a role for miR-151a’s in NSCLC EMT is emphasized by the recent finding that NSCLC cells featuring partial EMT (hybrid epithelial/mesenchymal phenotype) are endowed with higher cancer-initiating stem cell (CIC) plasticity and a significant shorter overall survival.^20^

Based on the significant decrease/loss of E-cadherin protein expression in our A549 cell studies, we were surprised to find that NSCLC patient specimens showed E-cadherin staining at varying degrees, even in the presence of significant miR-151a expression, as determined by *in situ* hybridization. This is likely a result of heterogeneity of the tumor. However, comparison of normal epithelial cell tissue (bronchiole) to tumor epithelial cells (NSCLC) within the same slide, indicates that NSCLC cells are characterized by high miR-151a expression and lower E-cadherin expression as compared to bronchiole tissue, which would be in agreement with our *in vitro* results.

In conclusion, our results strongly suggest that miR-151a functions as an oncomiR in NSCLC pathogenesis, by promoting tumor cell growth and inducing partial EMT, through the regulation of key gene products including E-cadherin, Fibronectin and Slug. Furthermore, we have determined that E-cadherin, a direct and functional target of miR-151a, can potently inhibit NSCLC cell migration and the transition to a mesenchymal-like cell phenotype, indicating that miR-151a-induced E-cadherin repression is a primary mechanism by which miR-151a enhances partial EMT of NSCLC. The identification of E-cadherin as a primary target of oncomiR-151a provides new insights into the understanding of the complex processes of partial NSCLC EMT, and may facilitate the development of potential therapeutics against NSCLC.

## Material and methods

### Patient samples

Formalin-fixed, paraffin embedded surgical specimens from 52 LAC (NSCLC) patients, for more details.^25^ The study was approved by the Regional Ethical Committee (Permission No.: 1-10-72-20-14) and all experiments were conducted in accordance with this approval.

### Cell culture, plasmids and treatments

Cells were incubated at 37 °C and 5% CO_2_ and routinely checked for mycoplasma contamination. Mouse lung endothelial cells (mLEC; C57-6011, Cell Biologics) were maintained in complete mEC media (M1168, Cell Biologics, Chicago, IL, USA) and 10% FBS (FB-02, Omega Scientific, Tarzana, CA, USA). Human lung EC (hLEC; #3000, ScienCell, Carlsbad, CA, USA) were maintained on plates coated with 10 μg/ml fibronectin (F2006, Sigma-Aldrich, St. Louis, MO, USA) in EC media (1001, ScienCell). Human NSCLC cell lines A549 (CCL-185), HEK293T (CRL-3216), H23 (CRL-5800) and NCI-H1299 (CRL5803) from American Tissue Cell Culture (ATCC) were cultured in DMEM (25-501N, Genesee, San Diego, CA, USA) and RPMI (SH30027FS, ThermoFisher, Hamton, NH, USA) medium, HeLa cells (CCL-2, ATCC) in EMEM (SH3024401, Hyclone, Anaheim, CA, USA) respectively, with 10% FBS. Five ng/ml TGF-β was added to some cultures (Peprotech, Rocky Hill, NJ, USA, Cat 100-21). All cell lines were tested for mycoplasma contamination routinely.

Plasmids used: mutations were introduced into an E-cadherin pcDNA overexpression plasmid (45769, Addgene, Cambridge, MA, USA) using the GeneArt Site-directed Mutagenesis System (A13282, Life Technologies, Carlsbad, CA, USA).

### RNA extraction and RT-qPCR

For all cell lines, RNA extraction and RT-qPCR experiments were conducted as previously described.^22^ From each formalin-fixed, paraffin embedded patient sample, RNA was extracted from a 1 × 7 μm section using the miRNeasy FFPE kit (217504, Qiagen, Germantown, MD, USA). miR expression analysis was performed using the miRCURY LNA Universal RT microRNA PCR system (203301, Exiqon, Woburn, MA, USA), whereas mRNA expression analysis was performed using the High Capacity Reverse Transcriptase Kit (4368813, Life Technologies) and TaqMan PreAmp Master Mix kit (4384267, ThermoFisher) according to manufacturer’s protocol. All RT-qPCR was performed in technical cDNA and qPCR duplicates using either hsa-miR-103a-3p and hsa-miR-423-5p or IPO8 and PUM1 as reference genes, as they have previously been reported stably expressed in NSCLC.^25, 26^ All data was analyzed using NormFinder to ensure stability of the reference genes.^27^ For each sample, relative quantities were calculated as 2^−ΔCt^ and determined as the average relative quantities in the cDNA synthesis duplicates.

### *In situ* hybridization and immunohistochemistry

For *in situ* hybridization analysis of miR-151a expression, a 5′- and 3′-double digoxigenin-labeled miRCURY LNA microRNA Detection Probe (612499-360, Exiqon) was used following manufacturer’s protocol. Scrambled probe and U6 were included as controls. Briefly, the deparaffinized, proteinase K-digested and dehydrated sections were hybridized with 40 nM double-DIG LNA hsa-miR-151a-5p probe for 60 min at 50 °C. After a series of stringent washes with saline-sodium citrate buffer, sections were blocked then incubated with 1:125 dilution of anti-DIG Fab fragments conjugated to alkaline phosphatase (11093274910, Roche Diagnostics, Indianapolis, IN, USA). After 30 min, the anti-DIG/AP was repNSCLCed with fresh reagent and incubated for an additional 30 min. The signal was detected using freshly prepared NBT/BCIP AP substrate (11-681-451-001, Roche Diagnostics). After 60 min at 30 °C, fresh AP substrate was added and incubated for 60 min. Finally, slides were counterstained with Nuclear Fast Red (H-3403, Vector laboratories, Burlingame, CA, USA). Immuhistochemical staining was performed routinely using the VENTANA BenchMark XT staining system (Roche Diagnostics) with antibodies against E-cadherin (790-4497, Ventana, Roche Diagnostics) and cytokeratin 7 (790-4462, Ventana, Roche Diagnostics) according to manufacturer’s protocol.

### Transfection and transduction of miRs

Transient transfection: 20 nM miRIDIAN microRNA mimics or inhibitors from GE Dharmacon, Lafayette, CO, USA (Control #2 (CN-002000-01-05), hsa-miR-151a-5p mimic (C-301086-01-0005), and hsa-miR-151a-5p Hairpin Inhibitor (IH-301086-02-0005)) using OptiMEM (31985070, ThermoFisher) and Lipofectamine RNAiMAX (13778150, ThermoFisher).

Stable transduction: VSV-G-pseudotyped lentiviral particles were made by transfecting 5.3 μg of pMD2-G (12259, Addgene), 9.7 μg of pCMV-DR8.74 (8455, Addgene) and 15 μg pCD510B-1 (miR Control), mZIP, pCD510B-1-miR-151a or mZIP-anti-miR-151a into 293T cells using Lipofectamine LTX (15338030, ThermoFisher).^22^ Transduced cells were selected and maintained using 10 μg/ml puromycin or 10 μg/ml blasticidin.

### Cell proliferation and colony formation assays

Cells were plated at low density (6 well plates at 2 × 10^4^ cells/well) in triplicate and counted or plated in 6 well plates at 200 cell/well in triplicate, cultured for 2 weeks, stained (0.1% crystal violet solution (C0775, Sigma), 0.3% acetic acid, 99.6% ethanol) and the area of colonies were determined.

### *In vitro* migration and invasion assays

Cells were pre-treated with 10 μg/ml mitomycin c (BP25312, Fisher Scientific, Hampton, NH, USA) for 2 h. Confluent cells were scratched and imaged after 6 or 14 h and the percent healed was calculated.

Transwell migration assay: 6.5 mm transwells with (8 μm) inserts (3464, Corning, Corning, NY, USA) were coated for 2 h with 10 μg/ml fibronectin (F2006, ThermoFisher). Migrated cells were fixed, stained with DAPI and counted.

Invasion assay: 8 μm PET inserts coated with Matrigel (354480, Corning) were used. After 12 h invasion was determined as in transwell experiments.

### Immunofluorescent staining

Cells were plated on gelatin-coated coverslips, fixed in 4% paraformaldehyde (Sigma-Aldrich), incubated in blocking buffer (1% bovine serum albumin, 0.3% Triton X-100 (ThermoFisher) in PBS), stained with 10 μg/ml goat anti-human E-cadherin antibody (AF648, R&D Systems, Minneapolis, MN, USA) followed by Donkey anti-Goat IgG secondary antibody conjugated to Alexa Flour 488 (A11055, ThermoFisher). Coverslips were mounted on slides with VectaSheild with DAPI (H-1200, Vector Laboratories) and cells imaged at 63x on a Zeiss spinning disk confocal microscope.

### Western blot

Cells were lysed in RIPA buffer (89901, ThermoFisher) with inhibitor cocktail (PI78410, ThermoFisher). 4x LDS sample buffer (NP0008, ThermoFisher) was used, samples boiled at 95 °C for 10 min. NuPAGE Novex 4-12% Bis–Tris Protein Gels (NP0335, ThermoFisher Scientific), PVDF membranes, Blocking (PBST 5% nonfat milk), primary antibodies E-cadherin (3195S, Cell Signaling, Danvers, MA, USA) or tubulin (ab4074, abcam, Cambridge, MA, USA), secondary antibody (HRP-linked anti-rabbit IgG antibody, 7074S, Cell Signaling) and Pierce ECL Western Blotting Substrate (32106, ThermoFisher) and Bio-Rad, Hercules, CA, USA ChemiDoc XRS+ System were used for protein expression development.

### Luciferase assay

Wildtype or mutated E-cadherin sequences from the coding region sequence (CRS) were cloned into a dual luciferase reporter plasmid (pEZX-MT05, Genecopoeia, Rockville, MD, USA). 3 × 10^5^ HeLa cells were forward-transfected with 0.8 μg reporter plasmid and 20 nM control mimic or miR-151a mimic with Attractene transfection reagent (301005, Qiagen) according to the manufacturer’s instructions. Relative *Gaussia* luciferase and secreted alkaline phosphatase were determined with the Secrete-Pair Dual Luminescence Assay Kit (SPDA-D010, Genecopoeia) on a Tecan Infinite F200 microplate reader.

### Argonaute RNA immunopurifications

Immunopurification of Argonaute from A549 cell extracts was performed using the 4F9 antibody (4F9, Santa Cruz Biotechnology, Dallas, TX, USA) as described previously.^22^ Results were normalized to their inputs and shown as ‘corrected’ values as a proxy for Ago immunopurification efficiency.

### Analysis of published data using The Cancer Genome Atlas database

The data was retrieved from https://gdc-portal.nci.nih.gov/. All hsa-mir151a expression values from the 45 patients with paired samples, normal lung tissue (sample type 11A) and tumor tissue (sample type 01A) were collected.

### Statistical analysis

Student’s *t*-tests were used to calculate two-tailed *P*-values and data are displayed as mean±s.e.m. of technical or independent biological replicates, (*n*) as indicated.
