# Supplementary material for: miR-151a induces partial EMT by regulating E-cadherin in NSCLC cells
Source: Oncogenesis. 2017 Jul 31;6(7):e366–. doi: 10.1038/oncsis.2017.66 (PMC5541717; doi:10.1038/oncsis.2017.66)

Supplementary Figure S1: miR-151a and miR-34a expression in non-malignant human tissue. miR-151a and miR-34a expression levels were determined in non-malignant human tissues (lung (n=10), adrenal gland (n=4) and brain (n=4) (n=2 technical cDNA replicates, 2 technical RT-qPCR per sample).

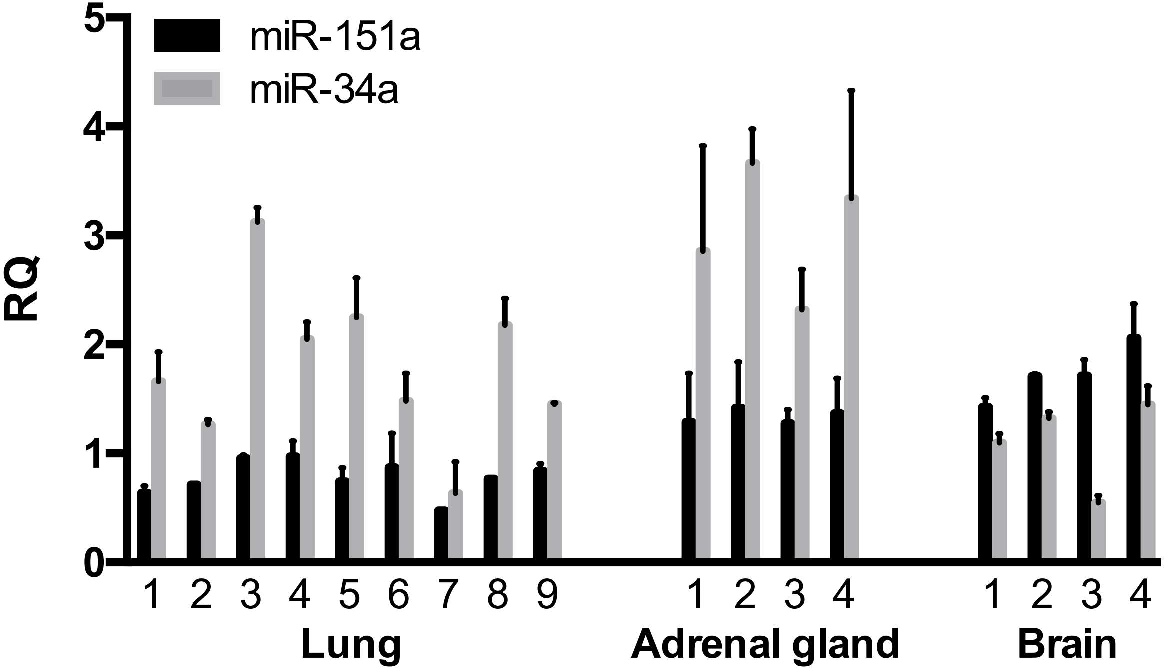

Supplement: Supplementary Figure S1 [file oncsis201766x1.pdf]
