# Supplementary material for: miR-151a induces partial EMT by regulating E-cadherin in NSCLC cells
Source: Oncogenesis. 2017 Jul 31;6(7):e366–. doi: 10.1038/oncsis.2017.66 (PMC5541717; doi:10.1038/oncsis.2017.66)

Supplementary Figure S2: miR-151a expression is enhanced in primary NSCLC. miR-151a expression levels were characterized in three primary NSCLC (LACs) by *in situ* hybridization, showing that miR-151a is highly expressed in primary lung tumor ("T") as compared to tumor-adjacent normal lung ("N") tissue. Representative images from each patient are shown. Images from patient 1 were included in Figure 1.

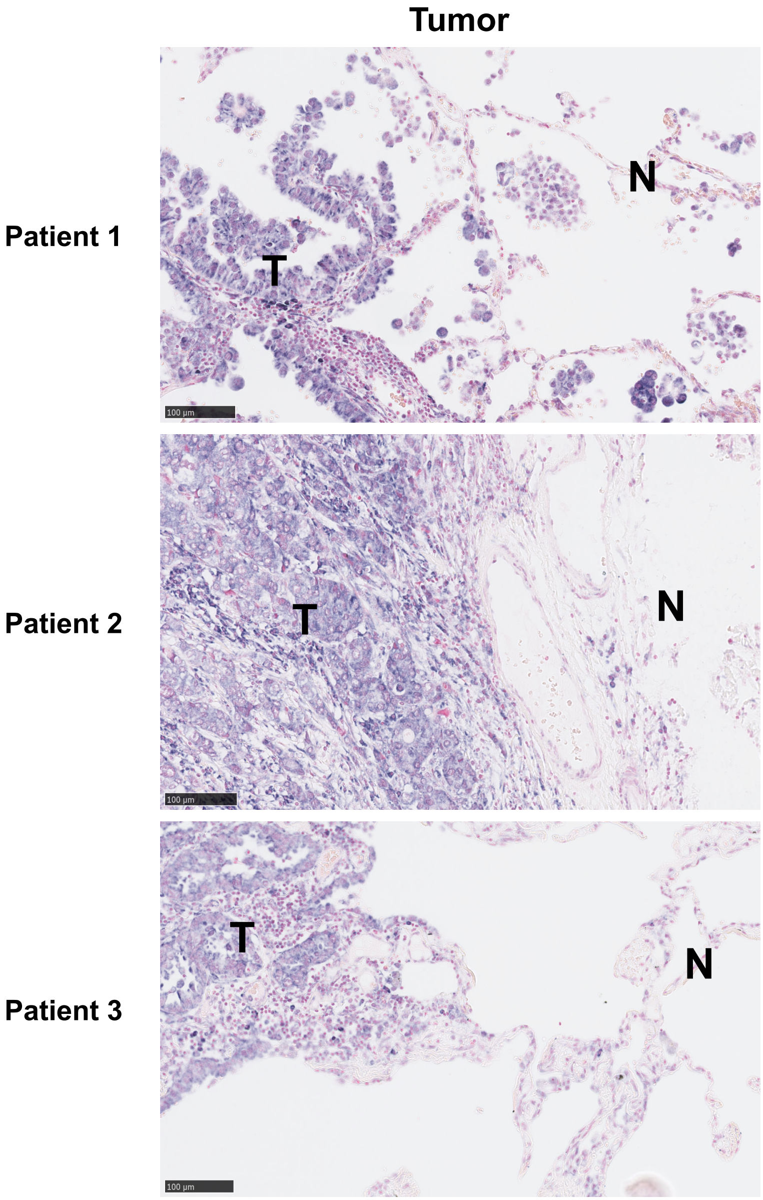

Supplement: Supplementary Figure S2 [file oncsis201766x2.pdf]
