# Supplementary material for: miR-151a induces partial EMT by regulating E-cadherin in NSCLC cells
Source: Oncogenesis. 2017 Jul 31;6(7):e366–. doi: 10.1038/oncsis.2017.66 (PMC5541717; doi:10.1038/oncsis.2017.66)

Supplementary Figure S3: Verification of miR-151a over-expression and neutralization of miR-151a in NSCLC cells. The relative quantity (RQ) of miR-151a (left panel) and RhoGDIA mRNA (right panel) was determined by RT-qPCR in A549s stably expressing miR-CTL, miR-151a or anti-miR-151a (miR-151a: n=3, ****p<0.0001 (miR-151a), ****p<0.0001 (anti-miR-151a), RhoGDIA: n=3, ****p<0.0001 (miR-151a), *p=0.0107 (anti-miR-151a)). Shown as mean ± SEM. Statistical significance was assessed using unpaired student's t-test with p-values 0.05 considered significant.

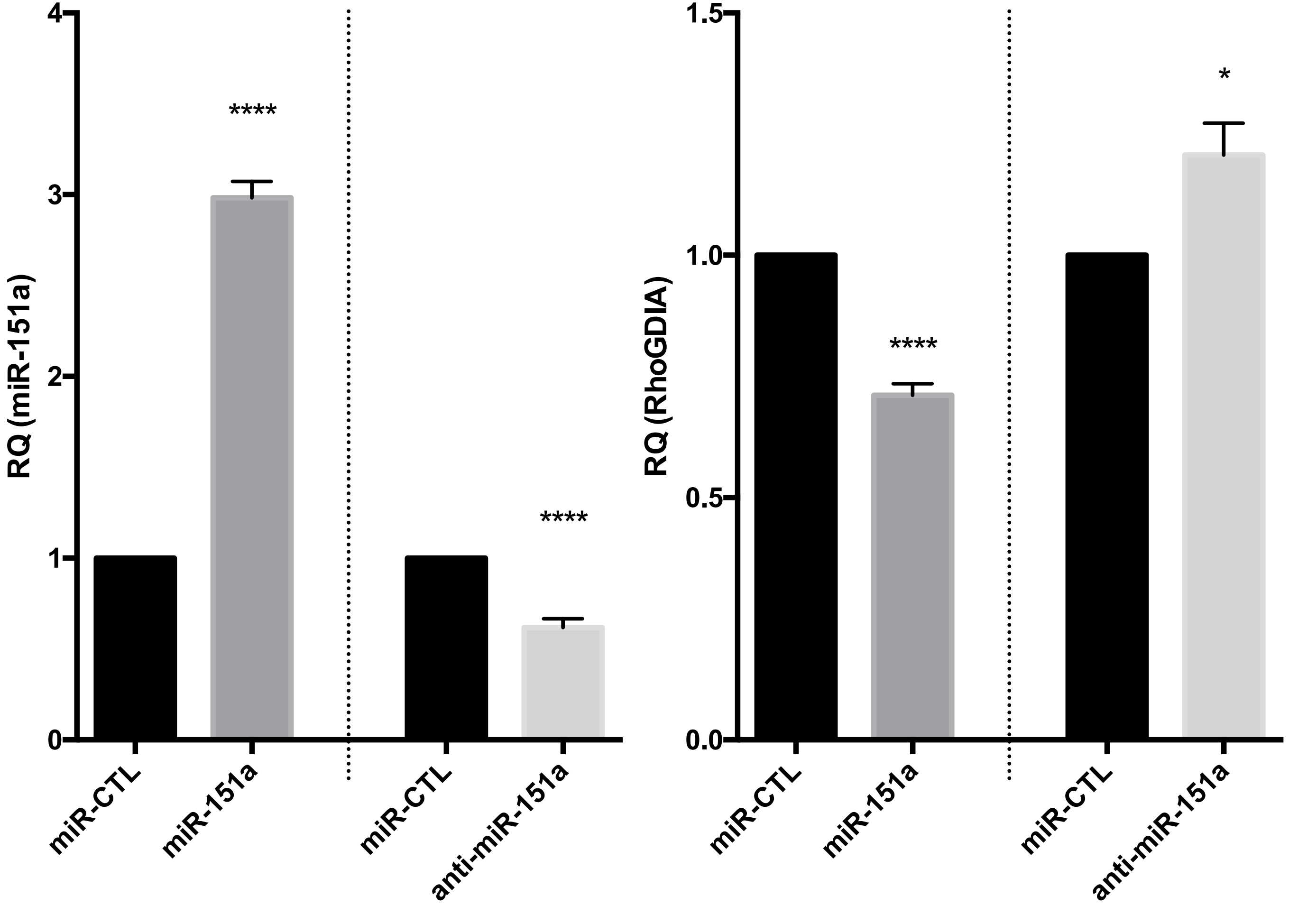

Supplement: Supplementary Figure S3 [file oncsis201766x3.pdf]
