# Supplementary material for: miR-151a induces partial EMT by regulating E-cadherin in NSCLC cells
Source: Oncogenesis. 2017 Jul 31;6(7):e366–. doi: 10.1038/oncsis.2017.66 (PMC5541717; doi:10.1038/oncsis.2017.66)

Supplementary Figure S4: miR-151a specifically affects NSCLC growth. Growth analysis of A549s stably **(A)** or transiently **(B)** expressing miR-CTL, miR-151a or anti-miR-151a counted 96 hours after plating (n=3, ***p*=0.005 (transduced miR-151a), ***p*=0.0045 (transduced anti-miR-151a), **p*=0.0472 (transfected miR-151a), **p*=0.0366 (transfected anti-miR-151a). **(C)** A549s stably expressing miR-CTL, anti-miR-151a or both miR-151a and anti-miR-151a counted 96 hours after plating (n=3, *****p*=0.0001 (anti-miR-151a)). **(D)** hLECs stably expressing miR-CTL miR-151a, or anti-miR-151a counted 96 hours after plating (n=1). All graphs are shown as mean ± SEM at the 96 hr time point. Statistical significance was assessed using unpaired student's t-test with *p*-values 0.05 considered significant.

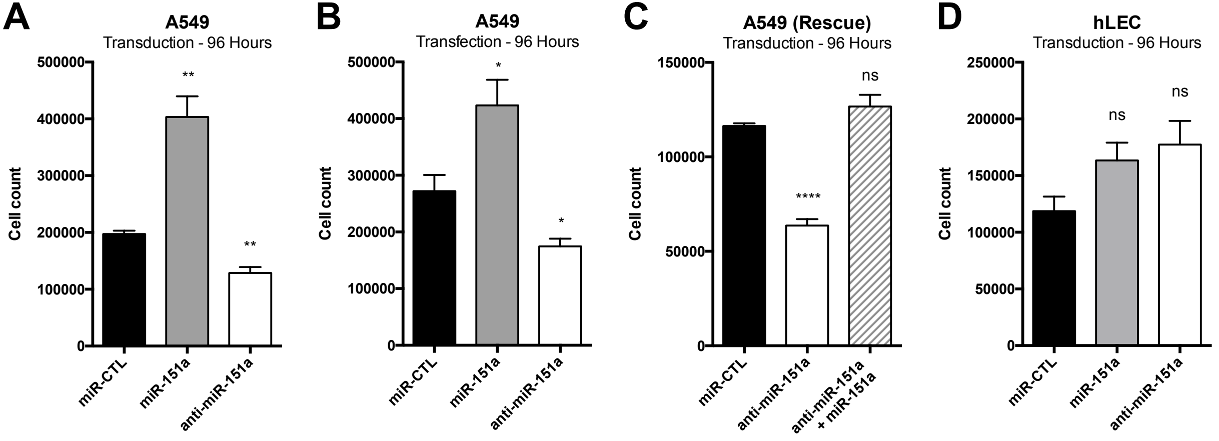

Supplement: Supplementary Figure S4 [file oncsis201766x4.pdf]
