# Supplementary material for: miR-151a induces partial EMT by regulating E-cadherin in NSCLC cells
Source: Oncogenesis. 2017 Jul 31;6(7):e366–. doi: 10.1038/oncsis.2017.66 (PMC5541717; doi:10.1038/oncsis.2017.66)

Supplementary Figure S5: miR-151a affects the migratory properties of NSCLC cells. (A) Growth analysis counting A549s untreated and pre-treated with mitomycin c for 2 hrs (n=1, *p=0.0248 at 12 hr). The migratory potential of **(B)** A549 cells transfected or **(C)** H23 cells transduced and **(D)** H1299 transduced with miR-CTL, miR-151a, or anti-miR-151a were assessed by wound healing assays. Confluent monolayers were treated with mitomycin c for 2 hrs and then scratched with a pipette tip. Wound areas were measured at time 0 and 6 hrs. (transfected A549s n=3, *** p=0.0005, transduced H23 n=3, *p=0.0304, transduced H1299 n=3, *p=0.0499). Scale = 500 m. Graphs are shown as mean ± SEM and statistical significance was assessed using unpaired student's t-test with p-values 0.05 considered significant.

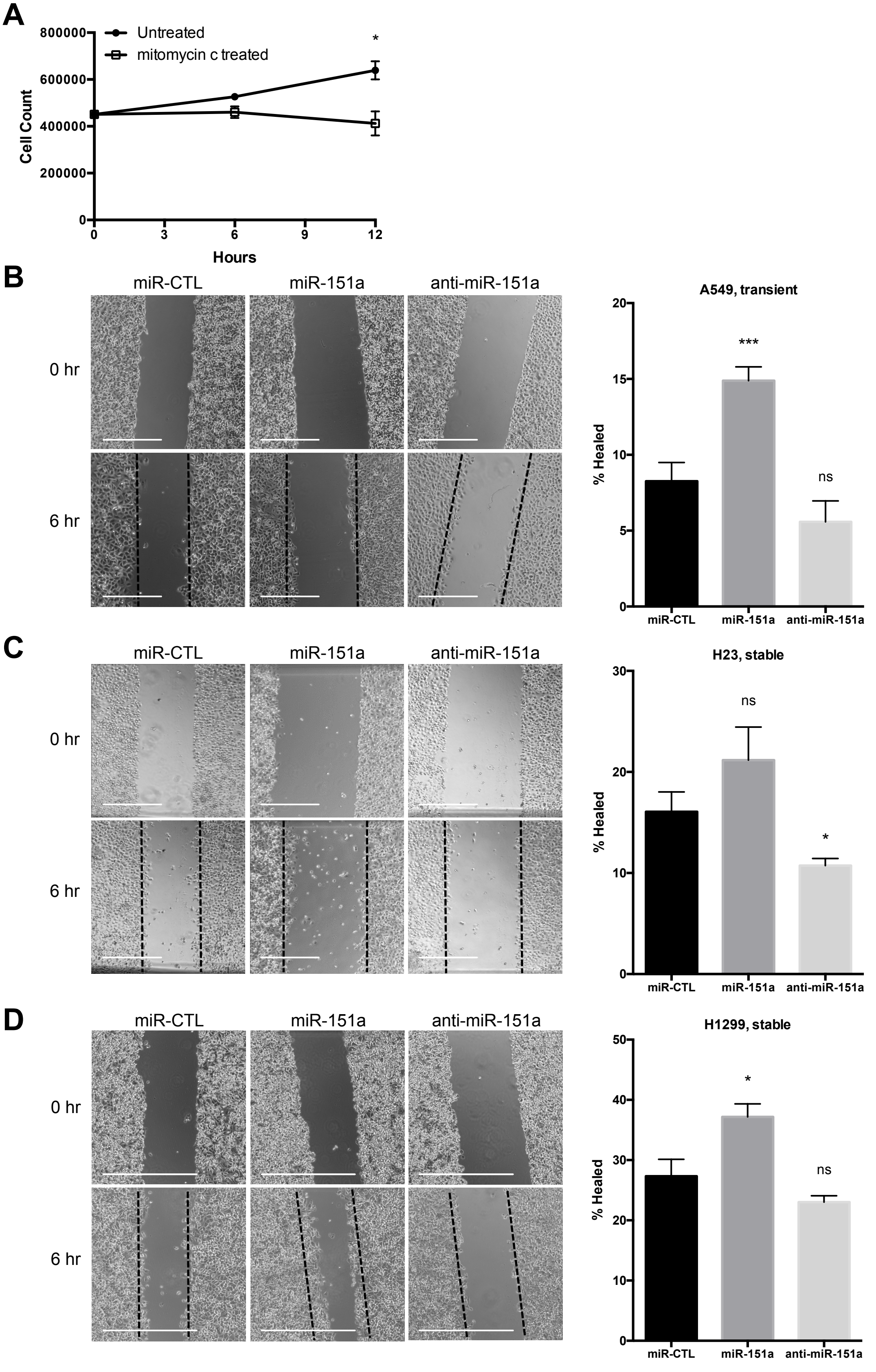

Supplement: Supplementary Figure S5 [file oncsis201766x5.pdf]
