# Supplementary material for: miR-151a induces partial EMT by regulating E-cadherin in NSCLC cells
Source: Oncogenesis. 2017 Jul 31;6(7):e366–. doi: 10.1038/oncsis.2017.66 (PMC5541717; doi:10.1038/oncsis.2017.66)

Supplementary Figure S6: miR-151a induces a mesenchymal-like phenotype in NSCLC. Images of A549 cells stably expressing miR-151a or anti-miR-151a. Bright field (first column), GFP expression (second column) and overlay (third column). Scale = 500 m. The relative proportion of mesenchymal, epithelial, or undefined cells were quantified and shown as pie charts.

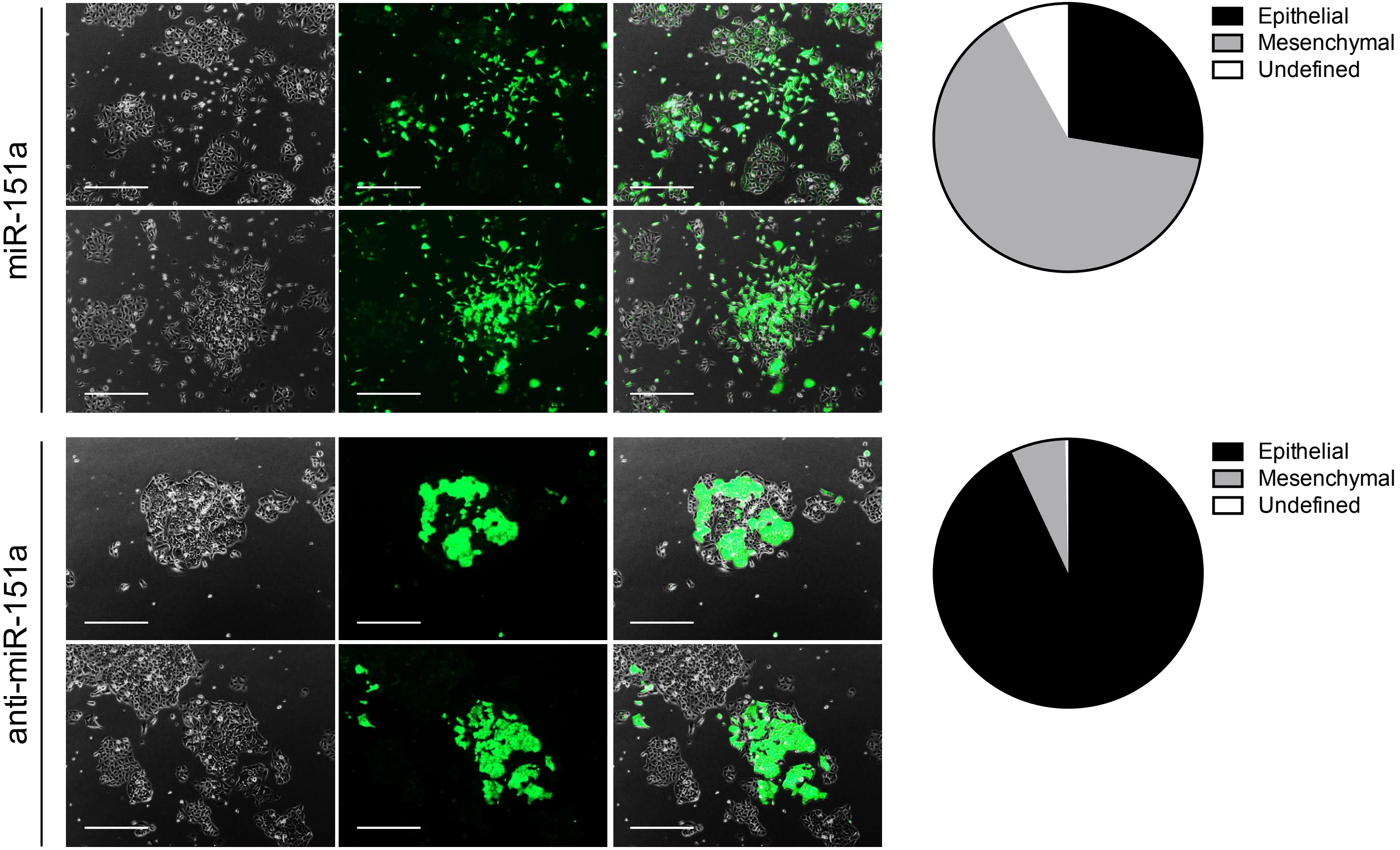

Supplement: Supplementary Figure S6 [file oncsis201766x6.pdf]
