# Supplementary material for: miR-151a induces partial EMT by regulating E-cadherin in NSCLC cells
Source: Oncogenesis. 2017 Jul 31;6(7):e366–. doi: 10.1038/oncsis.2017.66 (PMC5541717; doi:10.1038/oncsis.2017.66)

Supplementary Figure S7: miR-151a induces a mesenchymal-like phenotype in NSCLC. Bright field and GFP images of A549 cells untreated, treated with TGF-, or stably transduced with miR-CTL, miR-151a, or anti-miR-151a. Column 1: bright field and column 2: GFP expression (Scale = 400 m).

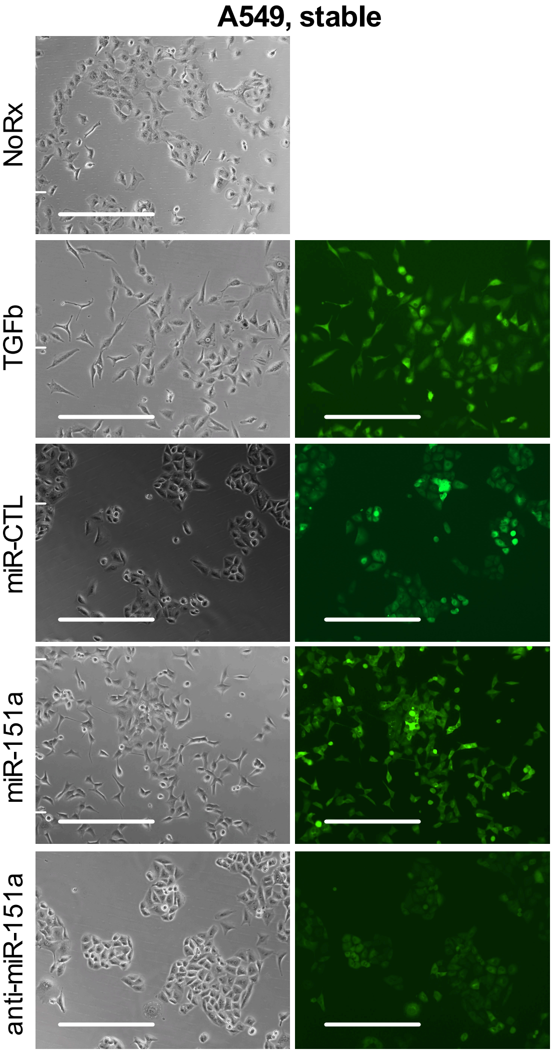

Supplement: Supplementary Figure S7 [file oncsis201766x7.pdf]
