# Supplementary material for: miR-151a induces partial EMT by regulating E-cadherin in NSCLC cells
Source: Oncogenesis. 2017 Jul 31;6(7):e366–. doi: 10.1038/oncsis.2017.66 (PMC5541717; doi:10.1038/oncsis.2017.66)

Supplementary Figure S8: Uncropped western blots. (A) Uncropped version of immunoblot shown in Figure 5C. **(B)** Uncropped version and quantification of immunoblot shown in Figure 7A.

A

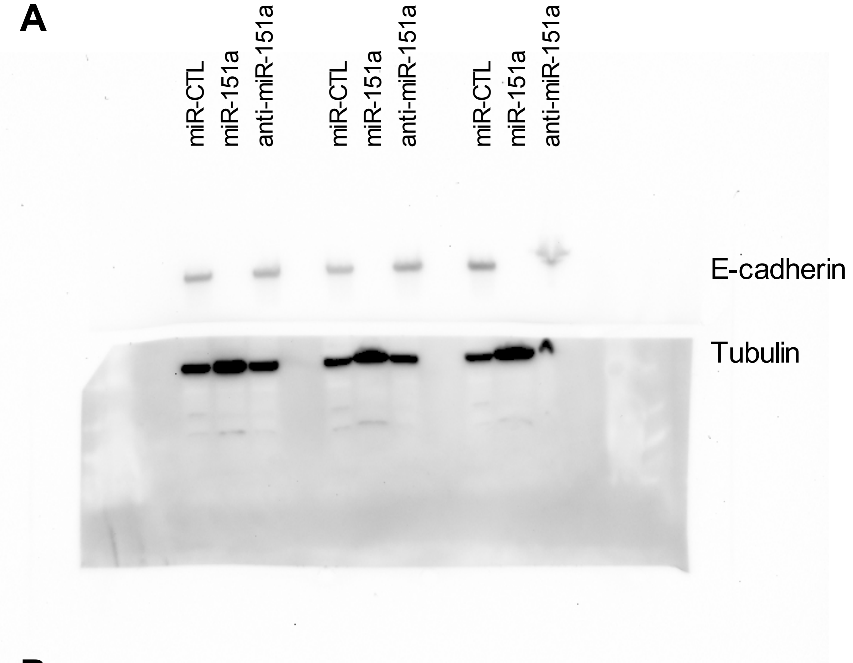

B

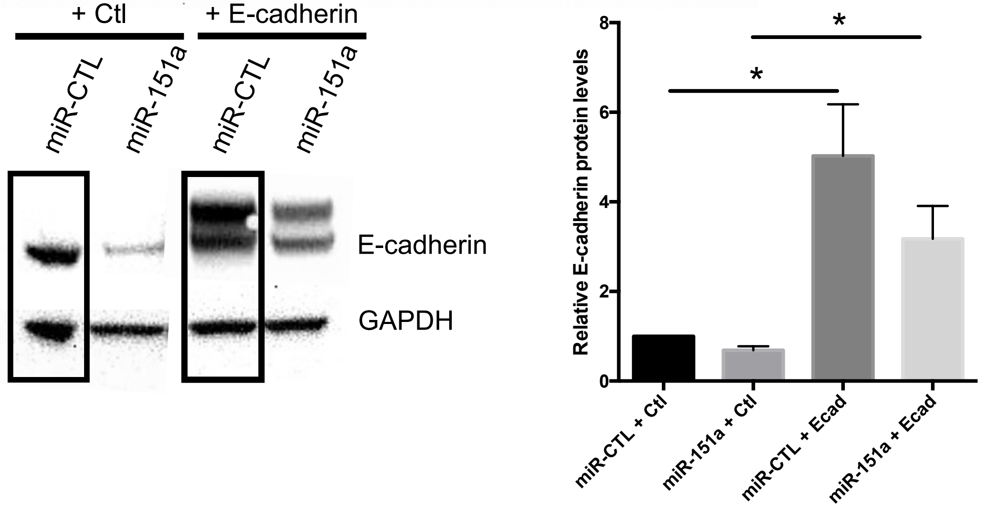

Supplement: Supplementary Figure S8 [file oncsis201766x8.pdf]
