# Supplementary material for: miR-151a induces partial EMT by regulating E-cadherin in NSCLC cells
Source: Oncogenesis. 2017 Jul 31;6(7):e366–. doi: 10.1038/oncsis.2017.66 (PMC5541717; doi:10.1038/oncsis.2017.66)
Supplement: Supplementary Figure S9 [file oncsis201766x9.pdf]

Supplemental Figure S9: E-cadherin and miR-151a correlation and miR-151a binding site conservation in E-cadherin mRNA. (A) The correlation between miR-151a and E-cadherin expression in NSCLC patient samples. The relative expression levels of miR-151a and E-cadherin were determined in 52 primary NSCLC samples by RT-qPCR. The potential correlation between miR-151a and E-cadherin expression was investigated by linear regression. The Line of Best Fit is indicated in black with grey dotted lines showing the 95% confidence interval (**p**=0.156, R2 = 0.0398). **(B)** mRNA sequence alignment of Homo sapiens (Z13009), Mouse (X06115), Leopard (XM_019455447), Gorilla (XM_004057872), Giant Panda (XM_002923207), and Cat (XM_003998189) as well as hsa-miR-151a (seed sequence, motif 1 to 3). Blue indicates homology between n≥5, green represents homology between n≥4 and orange represents homology between n≥ 3 species.

A

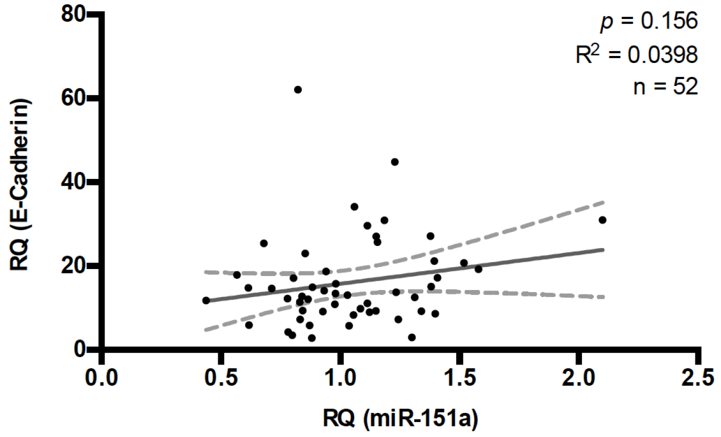

B

```
                Cat    (149)  CUCCUGCUGCUGCUACAGGUCGCAUCAGGGCUCUGCCAGGAGCCC
        Giant panda    (164)  CUCCUGCUGCUGCUACAGGUCUCAUCAUGGCUAUGCCAGGAGCCG
            Gorilla    (216)  CUGCUGCUGCUGCUGCAGGUUUCCUCUUGGCUCUGCCAGGAGCCG
            Leopard    (160)  CUCCUGCUGCUGCUACAGGUCACAUCAGGGCUCUGCCAGGAGCCC
              Mouse     (99)  CUCCUGCUCCUGCUGCAGGUCUCCUCAUGGCUUUGCCAGGAGCUG
       Homo sapiens    (125)  CUGCUGCUGCUGCUGCAGGUCUCCUCUUGGCUCUGCCAGGAGCCG
 motif 1 E-Cadherin           --------------------CUCCUC-------------------

          Consensus    (226)  CU CUGCUGCUGCU CAGGUCUC UC UGGCUCUGCCAGGAGCC
                              271                                         315


                Cat    (512)  GAAACCCAGACAGAAGUGCUCACAUUUCCCAACUCCAAGCAUGGUCUUAG
        Giant panda    (533)  GAAACCCAGACAGAAGUGCUCACAUUUCCCAACUCCCAGCGUGGUCUCAG
            Gorilla    (585)  GGAAUCCAAGCAGAAUUGCUCACAUUUCCCAACUCCUCUCCUGGCCUCAG
            Leopard    (523)  GAAACCCAGACAGAAGUGCUCACAUUUCCCAACUCCCAGCAUGGUCUUAG
              Mouse    (474)  GAAUCCAACCCAGAGCUGCUCAUGUUUCCCAGCGUGUACCCAGGUCUCAG
       Homo sapiens    (494)  GGAAUCCAAGCAGAAUUGCUCACAUUUCCCAACUCCUCUCCUGGCCUCAG
 motif 2 E-Cadherin           --------------------------------CUCCUC------------

          Consensus    (601)  G AA CCA  CAGAA UGCUCACAUUUCCCAACUCC   C UGG CU AG
                              651                                              700


                Cat   (2657)  UUAAACUCCUCGGAAUCAGACCGAGAUCAGGACUAUGACUACCUG
        Giant panda   (2678)  UUGAACUCCUCAGAGUCAGACCAAGACCAGGACUAUGACUACCUG
            Gorilla   (2727)  CUGAACUCCUCAGAGUCAGACAAAGACCAGGACUAUGACUACUUG
              Mouse   (2616)  CUGAACUCCUCUGAGUCGGAUCAGGACCAGGACUACGAUUAUCUG
            Leopard   (2668)  UUAAACUCCUCGGAAUCAGACCGAGAUCAGGACUAUGACUACCUG
       Homo sapiens   (2636)  CUGAACUCCUCAGAGUCAGACAAAGACCAGGACUAUGACUACUUG
 motif 3 E-Cadherin           -----CUCCUC----------------------------------

          Consensus   (2746)   UGAACUCCUC GAGUCAGAC  AGA CAGGACUAUGACUAC UG
                              2791                                        2835
```

Homology between species ≥ 5
Homology between species ≥ 4
Homology between species ≥ 3
