# Supplementary material for: miR-151a induces partial EMT by regulating E-cadherin in NSCLC cells
Source: Oncogenesis. 2017 Jul 31;6(7):e366–. doi: 10.1038/oncsis.2017.66 (PMC5541717; doi:10.1038/oncsis.2017.66)
Supplement: Supplementary Tables [file oncsis201766x10.pdf]

SUPPLEMENTARY TABLES

Supplementary Table S1: Clinical characteristics for the NSCLC cohort. Formalin-fixed, paraffin embedded (FFPE) surgical resections from 52 lung adenocarcinoma (LAC) patients were included in this study. The material includes 52 primary lung adenocarcinomas and 26 paired distant metastases (22 brain and 4 adrenal gland). Tumor-adjacent, normal lung tissue from 10 patients was included as controls.

Clinical Characteristics	NSCLC Cohort
**Patients (n)**	
Cases	52
Cases with paired distant metastases	24
**Histological subtype (n)**	
Adenocarcinoma	52
Squamous cell carcinoma	0
Adenosquamous cell carcinoma	0
Large cell carcinoma	0
Sarcomatoid carcinoma	0
**Gender (n (%))**	
Male	22 (42.3%)
Female	30 (57.7%)
**Age (years)**	
Min-Max (Average)	38-76 (61.7)
**TNM Classification (n (%))**	
T1	18 (34.7%)
T2	32 (61.5%)
T3	0 (0%)
T4	1 (1.9%)
T Unknown	1 (1.9%)
N0	33 (63.5%)
N1	8 (15.4)
N2	9 (17.3%)
N3	0 (0%)
N Unknown	2 (3.8%)
M0	26 (50.0%)
M1	26 (50.0%)
**Smoking status (n (%))**	
Current Smoker	34 (65.4%)
Previous Smoker	16 (30.8%)
Unknown	2 (3.8%)
**Tumor content (%)**	
Tumors, Min-Max (Average)	5-80% (33.2%)
Metastases, Min-Max (Average)	5-90% (68.7%)
